# Hydrothermal Corrosion Resistance of Reaction-Bonded SiC Ceramic: Synergistic Enhancement by Homogeneous MoSi_2_ Distribution and Residual Silicon Reduction

**DOI:** 10.3390/ma19102039

**Published:** 2026-05-13

**Authors:** Shuaixu Chun, Haifeng Nie, Xiaoyang Guo, Tihao Cao, Quanxing Ren, Qing Sun, Zhengren Huang, Qing Huang, Yinsheng Li

**Affiliations:** 1 College of Materials Science and Engineering, Zhejiang University of Technology, Hangzhou 310014, China; chunshuaixu@nimte.ac.cn (S.C.); qingsun@zjut.edu.cn (Q.S.); 2 Zhejiang Key Laboratory of Data-Driven High-Safety Energy Materials and Applications, Ningbo Key Laboratory of Special Energy Materials and Chemistry, Ningbo Institute of Materials Technology and Engineering, Chinese Academy of Sciences, Ningbo 315201, China; nie_haifeng@126.com (H.N.); guoxiaoyang@nimte.ac.cn (X.G.); zhrhuang@nimte.ac.cn (Z.H.); huangqing@nimte.ac.cn (Q.H.); 3 Qianwan Institute of CNITECH, Ningbo 315336, China

**Keywords:** reaction-bonded silicon carbide, MoSi_2_, hydrothermal corrosion, molten salt synthesis, residual silicon

## Abstract

Reaction-bonded SiC (RBSC) ceramics exhibit limited hydrothermal corrosion resistance due to the presence of residual silicon. This study presents a strategy to enhance the corrosion resistance of RBSC through homogeneous incorporation of MoSi_2_ and concurrent reduction in residual silicon content. Three material systems were fabricated via reactive melt infiltration: conventional RBSC with a SiC/C preform (SC), a SiC–MoSi_2_ composite incorporating commercial Mo_2_C powder via physical mixing (MC), and a SiC–MoSi_2_ composite derived from a Mo_2_C/C precursor synthesized by a molten salt method (MS). The Mo_2_C/C composite synthesized at 1150 °C exhibited fine, uniformly distributed Mo_2_C particles coated on carbon black, contrasting with the agglomerated distribution in commercial Mo_2_C mixtures. During reactive sintering at 1600 °C, Mo_2_C reacted with molten Si to form MoSi_2_, reducing residual Si content. Sample MS achieved the lowest residual Si (8.77 ± 0.45 vol.%), followed by MC (12.43 ± 0.86 vol.%) and SC (19.17 ± 1.01 vol.%). All samples achieved near-full densification (open porosity < 0.1%), with bulk densities of 2.96 ± 0.05, 3.03 ± 0.03, and 3.07 ± 0.03 g/cm^3^ for SC, MC, and MS, respectively. Microstructurally, MS displayed homogeneous MoSi_2_ dispersion, while MC showed partial MoSi_2_ aggregation, and SC contained continuous residual Si regions. Hydrothermal corrosion tests at 345 °C and 15 MPa for 9 days demonstrated that corrosion resistance followed the order MS > MC > SC. After 9 days, weight loss was 22.3970 ± 1.2059 mg/cm^2^ (SC), 17.6370 ± 0.8266 mg/cm^2^ (MC), and 15.4347 ± 0.7807 mg/cm^2^ (MS), with corrosion depths of 393.17 ± 27.46, 267.40 ± 24.44, and 224.60 ± 25.13 μm, respectively. The enhanced performance of MS arises from two synergistic factors: reduced residual Si minimizes large corrosion pores, while uniform distribution of MoSi_2_ facilitates the formation of a stable, dissolution-resistant composite oxide layer composed of MoO_3_ and SiO_2_, in which MoO_3_ restrains excessive dissolution of SiO_2_ through a pinning effect. These findings demonstrate that combining residual Si reduction with homogeneous MoSi_2_ incorporation via molten salt-synthesized precursors offers an effective strategy for improving hydrothermal corrosion resistance of reaction-bonded SiC-based materials for applications in high-temperature and high-pressure aqueous environments such as nuclear water reactors.

## 1. Introduction

Silicon carbide (SiC) is an advanced ceramic with exceptional comprehensive properties, including excellent high-temperature mechanical strength, oxidation resistance, corrosion resistance, and radiation resistance. It is widely used in high-end manufacturing, optoelectronics, nuclear energy, advanced composites, and other high-tech fields [1,2,3,4,5,6,7,8,9]. In particular, SiC exhibits superior neutron irradiation resistance, low neutron absorption cross-section, minimal mechanical degradation under irradiation, and low volume swelling, making it an ideal material for key components in nuclear reactors [10,11,12,13,14,15,16]. Consequently, SiC-based materials, especially SiC fiber-reinforced SiC composites (SiC*_f_*/SiC), have emerged as leading candidates for accident-tolerant fuel (ATF) cladding in pressurized water reactors (PWRs).

Among various fabrication routes for SiC ceramics, reaction-bonded silicon carbide (RBSC) prepared via reactive melt infiltration (RMI) offers distinct advantages, including low processing cost, short fabrication cycles, near-net-shape capability with minimal shrinkage, and the ability to produce complex-shaped components [17,18,19,20,21]. During RMI, molten silicon infiltrates a porous SiC/C preform and reacts with carbon to form secondary β-SiC, resulting in nearly fully dense materials with open porosity approaching zero. However, an inherent limitation of this process is the unavoidable presence of residual free silicon, typically ranging from 15 to 20 vol.%, which remains unreacted in the final microstructure [22,23,24,25].

In PWR environments, fuel cladding materials are subjected to high-temperature and high-pressure water (typically ∼300–360 °C, ∼15 MPa) over extended service periods. Under such hydrothermal conditions, the residual silicon in RBSC is preferentially attacked, undergoing oxidation to form amorphous SiO_2_, which subsequently dissolves as soluble silicate species (e.g., H_2_SiO_3_ or Si(OH)_4_) [26,27,28]. This dissolution process creates interconnected pores and corrosion channels, allowing further ingress of the corrosive medium and accelerating degradation of the material. Therefore, improving the hydrothermal corrosion resistance of RBSC by alleviating the detrimental effects associated with residual silicon is critical to its application as nuclear fuel cladding in pressurized water reactors (PWRs).

One promising strategy to address this challenge is to convert the residual silicon into more chemically stable phases. Reported approaches for controlling residual silicon in the literature, including powder gradation optimization, CVD pyrolytic carbon coating, and carbon precursor infiltration pyrolysis (CPIP), primarily focus on improving mechanical properties and lack targeted design and optimization for hydrothermal corrosion resistance [29,30,31]. Molybdenum disilicide (MoSi_2_) is particularly attractive for this purpose due to several advantageous characteristics. Molybdenum possesses a low thermal neutron absorption cross-section, which is essential for maintaining neutron economy in nuclear reactors [32,33,34,35,36]. Furthermore, MoSi_2_ exhibits outstanding oxidation resistance even at elevated temperatures, attributed to the formation of a protective and self-healing SiO_2_ glass layer upon exposure to oxidizing environments [37,38]. Indeed, MoSi_2_ is widely employed in high-temperature heating elements and protective coatings for refractory metals, demonstrating its reliability under extreme thermal and corrosive conditions [39,40,41]. By introducing Mo-containing precursor like Mo_2_C into the RBSC preform, the infiltrated molten silicon can react with it to form MoSi_2_, thereby reducing the residual silicon content while simultaneously incorporating a more corrosion-resistant silicide phase.

The effectiveness of this strategy, however, depends critically on the homogeneity and fineness of the Mo_2_C distributed within the preform. Commercial Mo_2_C powders typically exhibit coarse particle sizes and broad size distributions. Moreover, the significant density disparity between Mo_2_C (ρ = 9.18 g/cm^3^) and carbon black (ρ = 1.8 g/cm^3^) readily leads to powder segregation and agglomeration during mixing. Such microstructural inhomogeneities inevitably result in the formation of coarse MoSi_2_ grains, uneven phase distribution, and increased amounts of unreacted residual silicon, all of which compromise the hydrothermal corrosion resistance of the final material. The molten salt synthesis method offers unique advantages for overcoming these limitations, enabling the preparation of fine, uniformly coated composite powders through a dissolution–reprecipitation mechanism in a liquid salt medium [42,43,44,45]. This approach has been successfully employed to synthesize various carbide and MAX phase powders with refined microstructures. Furthermore, compared with the complicated procedures of conventional coating processes, the molten salt method features simpler operation and a shorter flow, thereby showing greater potential for industrial applications [30].

In this study, three material systems were fabricated via reactive melt infiltration to systematically investigate the effect of MoSi_2_ incorporation and residual silicon reduction on hydrothermal corrosion resistance: (i) conventional RBSC with a SiC/C preform (SC); (ii) a SiC–MoSi_2_ composite incorporating commercial Mo_2_C powder via physical mixing (MC); and (iii) a SiC–MoSi_2_ composite derived from a Mo_2_C/C precursor synthesized by the molten salt method, subsequently mixed with SiC powder to form the preform (MS). The phase composition, densification behavior, microstructural evolution, residual silicon content, and hydrothermal corrosion performance at 345 °C and 15 MPa were systematically evaluated. Through comparative analysis, the underlying mechanisms by which homogeneous MoSi_2_ distribution and reduced residual silicon enhance hydrothermal corrosion resistance were elucidated. It should be noted that while this work focuses on monolithic SiC ceramics as a model system, the findings are expected to provide fundamental insights and technical guidance for advancing RMI-processed SiC*_f_*/SiC composites as potential accident-tolerant fuel cladding materials for future pressurized water reactors. This study ultimately validates the core hypothesis that uniformly distributed MoSi_2_ coupled with low residual silicon synergistically promotes the generation of a dense, stable, dissolution-resistant oxide scale, which efficiently restricts hydrothermal degradation and improves the service durability of SiC-based materials.

## 2. Experimental Procedure

### 2.1. Synthesis of Mo_2_C/C Composite Powder via Molten Salt Method

Carbon black (D_50_ = 20 nm, Qinhuangdao Yinuo High-Tech Material Development Co., Ltd., Qinhuangdao, China) and molybdenum powder (99.5% purity, D_50_ = 2 μm, Shanghai Macklin Co., Ltd., Shanghai, China) were used as starting materials. Sodium chloride (NaCl, ≥99.5 wt.%, Shanghai Aladdin Bio-Chem Technology Co., Ltd., Shanghai, China) and potassium chloride (KCl, ≥99 wt.%, Shanghai Aladdin Bio-Chem Technology Co., Ltd., Shanghai, China) served as the molten salt medium. The molten salt (NaCl:KCl = 1:1 in molar ratio) and raw materials (Mo:C = 1:6 in molar ratio) were uniformly mixed using an agate mortar and pestle at a mass ratio of 2:1 (salt:raw materials). The resulting mixture was poured into an alumina crucible and placed in an alumina tube furnace. The mixture was heated to target temperatures (850, 950, 1050, and 1150 °C) and held for 4 h under flowing argon to synthesize uniformly coated Mo_2_C on the carbon black surface via the molten salt method.

The as-obtained product, consisting of the synthesized powder and residual salts, was then immersed in deionized water and heated in a water bath at 70 °C with rotary stirring for 60 min to fully dissolve the salts, followed by vacuum filtration using a Büchner funnel and filter paper. This washing–filtration process was repeated three times to completely remove residual salts. Finally, the washed powders were dried in an oven at 100 °C for 24 h to obtain the Mo_2_C/C composite powder. The Mo/C molar ratio of 1:6 has been verified in preliminary experiments, and the Mo_2_C/C composite powder prepared by the molten salt method exhibits good reproducibility.

### 2.2. Fabrication of SiC Ceramic and SiC-MoSi_2_ Composites

For the preparation of reaction-bonded SiC ceramics and SiC–MoSi_2_ composites, the following raw materials were used: α-SiC powder (average particle size 0.5 μm, Qinhuangdao Yinuo High-Tech Material Development Co., Ltd., Qinhuangdao, China), silicon particles (particle size 1–3 mm, purity 99.99%, Qinhuangdao Yinuo High-Tech Material Development Co., Ltd., Qinhuangdao, China), commercial Mo_2_C powder (particle size 1–3 μm, purity 99.99%, Qinhuangdao Yinuo High-Tech Material Development Co., Ltd., Qinhuangdao, China), Mo_2_C/C composite powder synthesized via the molten salt method, carbon black, polyvinylpyrrolidone (PVP, Shanghai Aladdin Bio-Chem Technology Co., Ltd., Shanghai, China), and polyvinyl butyral (PVB, Aviation Grade, Sinopharm Chemical Reagent Co., Ltd., Shanghai, China).

Three different powder formulations were prepared: (1) Sample SC: 80 wt.% α-SiC and 20 wt.% carbon black; (2) Sample MC: 80 wt.% α-SiC, 7.8 wt% carbon black, and 12.2 wt.% commercial Mo_2_C powder; (3) Sample MS: 80 wt.% α-SiC and 20 wt.% Mo_2_C/C composite powder synthesized by the molten salt method. This formulation was designed to have the same nominal composition as Sample MC.

Each powder mixture was dispersed in ethanol with the addition of 2 wt.% PVP (based on total powder mass) as dispersant and 1 wt.% PVB as binder. The slurries were mixed in polyethylene bottles using zirconia balls as milling media on a roller mill for 8 h. After ball milling, the mixed slurries were dried using a vacuum rotary evaporator at 70 °C. The dried cakes were crushed and sieved, and the resulting powder mixtures were uniaxially dry-pressed in a stainless-steel mold, followed by cold isostatic pressing (CIP) at 200 MPa for 5 min.

The green compacts were subjected to a vacuum debinding treatment at 900 °C for 1 h to remove organic additives, and the green density was determined as follows: SC: 1.72 ± 0.04 g/cm^3^; MC: 1.76 ± 0.02 g/cm^3^; MS: 1.78 ± 0.03 g/cm^3^. Subsequently, the green bodies were infiltrated with molten silicon at 1600 °C for 2 h under vacuum (<30 Pa) in a graphite furnace. The resulting RB-SiC ceramic and RB-SiC–MoSi_2_ composites were ground and polished to a 1 μm finish using diamond suspensions.

### 2.3. Characterizations

The bulk density (*ρ*) and porosity (*p*) of the composite ceramics prepared by RMI were measured using Archimedes’ principle. Equations (1) and (2) were used to calculate *ρ* and *p*, respectively. The calculation methods for these values are as follows:

(1) $$\rho =\frac{m_{d}\rho_{w}}{m_{w}-m_{s}}$$

(2) $$P=\frac{m_{w}-m_{d}}{m_{w}-m_{s}}\times 100\%$$

where *ρ*_w_ is the density of deionized water, m_d_ is the dry mass of the sample, *m*_w_ is the saturated wet mass in air, and *m*_s_ is the suspended mass measured in water.

The residual silicon content in the ceramic samples was quantitatively determined by an alkali etching method. Previous studies have demonstrated that SiC and MoSi_2_ exhibit excellent alkali corrosion resistance [46,47,48]. Specifically, samples were first thinned to a thickness of 500 μm, then immersed in a 30 wt.% NaOH solution and heated in a water bath at 70 °C for three days. This mild alkali etching selectively removes residual Si without attacking other phases such as SiC or MoSi_2_ in the reactive sintered ceramic samples. The reaction between residual Si and NaOH solution can be described as Equation (3):

Si(s) + 2NaOH(aq) + H_2_O(l) = Na_2_SiO_3_(aq) + 2H_2_(g) (3)

After alkali etching, the volume fraction of residual Si was estimated from the mass change using the following formula:

(4) $$\mathrm{Vsi}=\frac{\rho (m_{1}-m_{2})}{\rho_{Si}\cdot m_{1}}\times 100\%$$

where *m*_1_ and *m*_2_ are the masses before and after alkali etching, *ρ* is the density of the reactive sintered sample, and *ρ*_Si_ is the density of silicon (2.33 g/cm^3^).

Hydrothermal corrosion tests were conducted in an autoclave (FGD, Dalian Tongchan Autoclave Manufacturing Co., Ltd., Dalian, China) using ultrapure water under conditions of 345 °C and 15 MPa for a total duration of 9 days. During the corrosion experiment, samples were taken out every 3 days to record mass changes. Three parallel tests were conducted for each sample. The mass change per unit area (∆*w*) was calculated as in Equation (5):

(5) $$∆w=\frac{m_{t}-m_{0}}{s}\times 100\%$$

where ∆*w* is the mass change per unit area (g/cm^2^), *m*_0_ is the initial mass (g), *m*_t_ is the mass after corrosion time t (g), and *s* is the surface area of the sample (cm^2^).

Phase identification was conducted by X-ray diffraction (XRD; D8 Advance, Bruker AXE, Karlsruhe, Germany). Microstructures and elemental composition were analyzed using field-emission scanning electron microscopy (FE-SEM; Quanta 250 FEG, FEI, Hillsboro, OR, USA) equipped with an energy-dispersive spectrometer (EDS). The chemical bonding states of elements in the ceramic samples were analyzed by X-ray photoelectron spectroscopy (XPS; Axis Supra, Kratos Analytical Ltd., Tokyo, Japan). The binding energy of all high-resolution XPS spectra was calibrated by referencing the characteristic C 1s peak of surface contamination carbon at 284.8 eV to eliminate the charging effect.

## 3. Results and Discussion

### 3.1. Synthesis and Characterization of Mo_2_C/C Composite Powder

**Figure 1:**
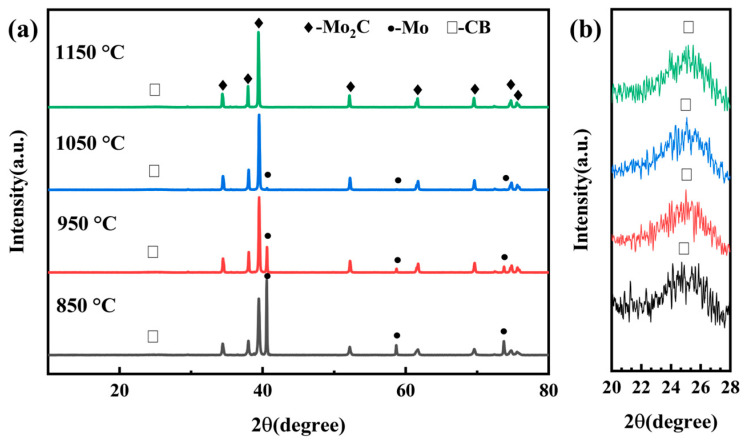
XRD patterns of the composite powders synthesized by the molten salt method at different temperatures (850, 950, 1050, and 1150 °C) for 4 h: (**a**) full spectrum; (**b**) locally magnified pattern.

Figure 1 shows the X-ray diffraction patterns of the powder products synthesized via the molten salt method at various temperatures (850–1150 °C) for 4 h. In all four samples, Mo_2_C is identified as the dominant crystalline phase. Broad weak peaks located at 2θ angles of 24–26° are also observed, which are characteristic of turbostratic carbon and correspond to the residual amorphous carbon black (Figure 1b). Additionally, diffraction peaks corresponding to metallic Mo appear in the products synthesized at 850–1050 °C, and their intensity decreases markedly with increasing synthesis temperature. When the temperature reaches 1150 °C, the Mo peaks disappear completely, indicating full conversion of the molybdenum reactant.

These results demonstrate that Mo_2_C can be effectively synthesized via the molten salt method, with the optimal temperature being 1150 °C, where the metallic Mo is completely consumed. The formation of Mo_2_C proceeds through the following reaction:

2Mo(s) + C(s) = Mo_2_C(s) (6)

The molten salt provides a liquid medium that facilitates reaction (6). The melting points of NaCl and KCl are approximately 801 °C and 770 °C, respectively, and their eutectic temperature is 657 °C. Since the synthesis temperatures employed in this study (850–1150 °C) exceed this eutectic point, the NaCl–KCl mixture forms a homogeneous liquid phase. This molten salt medium promotes the reaction kinetics through a dissolution–reprecipitation mechanism, accelerating the conversion of Mo and C into Mo_2_C.

As the synthesis temperature increases, the viscosity of the molten salt medium further decreases, enhancing mass transport and thereby improving the efficiency of the dissolution–reprecipitation process. At 1150 °C, the complete disappearance of metallic Mo diffraction peaks confirms that all Mo has been converted into Mo_2_C. Given that carbon black was present in excess relative to metallic Mo in the starting mixture, the final product obtained at this temperature is a composite powder consisting of Mo_2_C and residual carbon, as evidenced by the XRD patterns in Figure 1.

**Figure 2:**
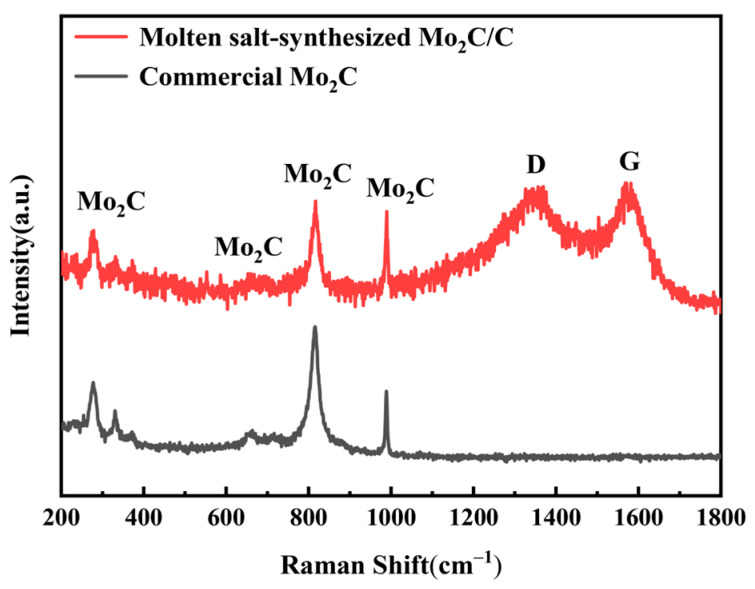
Raman spectra of commercial Mo_2_C and Mo_2_C/C composite.

The Raman spectra of the molten salt-synthesized Mo_2_C/C composite (Mo/C molar ratio = 1/6) and commercial Mo_2_C are shown in Figure 2. Both samples exhibit characteristic peaks of Mo_2_C at 284, 660, 818, and 989 cm^−1^, confirming the successful formation of the Mo_2_C phase. Only the Mo_2_C/C composite displays distinct D (~1345 cm^−1^) and G (~1583 cm^−1^) bands, which are attributed to residual carbon [49]. No signals corresponding to metallic Mo are detected, indicating that the metallic Mo precursor is completely converted to Mo_2_C, while the excess unreacted carbon forms the Mo_2_C/C composite.

**Figure 3:**
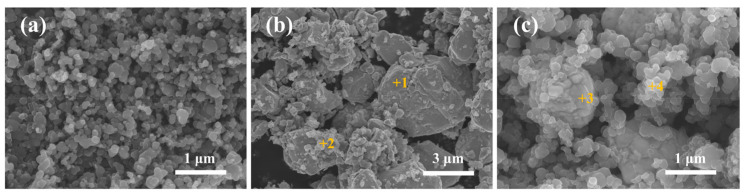
SEM images of (**a**) raw carbon black, (**b**) physical mixture of carbon black and commercial Mo_2_C powder, and (**c**) Mo_2_C/C composite powder synthesized by the molten salt method at 1150 °C.

Figure 3 compares the SEM morphologies of the raw carbon black, a physical mixture of carbon black and commercial Mo_2_C powder, and the Mo_2_C/C composite powder synthesized via the molten salt method at 1150 °C. The raw carbon black (Figure 3a) exhibits a uniform spherical nanoparticle morphology with an average diameter of approximately 20 nm. In the physical mixture (Figure 3b), both spherical nanoparticles (carbon black) and micron-sized particles (1–3 μm) are observed, but their distribution is inhomogeneous, with noticeable agglomeration of the carbon black nanoparticles. EDS point analysis (Table 1) confirms that the micron-sized particles correspond to Mo_2_C.

In contrast, the Mo_2_C/C composite powder synthesized by the molten salt method (Figure 3c) displays a homogeneous microstructure in which spherical carbon black nanoparticles are uniformly coated on the surface of ≈1 μm particles, with no visible agglomeration and excellent interfacial integration between the two components. EDS analysis (Table 1), together with the XRD results presented in Figure 1, confirms that the ≈1 μm particles are Mo_2_C.

These microstructural observations demonstrate that the Mo_2_C particles synthesized via the molten salt method are finer and more uniformly distributed within the carbon black matrix compared to the commercial Mo_2_C powder. This improved homogeneity arises from the unique synthesis mechanism: the molten salt provides a liquid medium in which Mo dissolves, migrates, and subsequently precipitates onto the carbon black surface, where it reacts according to Equation (6) to form Mo_2_C. Because this in situ reaction occurs directly on the carbon black surface—coupled with the relatively fine starting Mo particle size (≈2 μm) and the mild reaction conditions of the molten salt process—a highly uniform Mo_2_C/C composite powder with finer Mo_2_C particles is obtained [50].

### 3.2. Phase Composition and Microstructure of Reactive Sintered Composites

**Figure 4:**
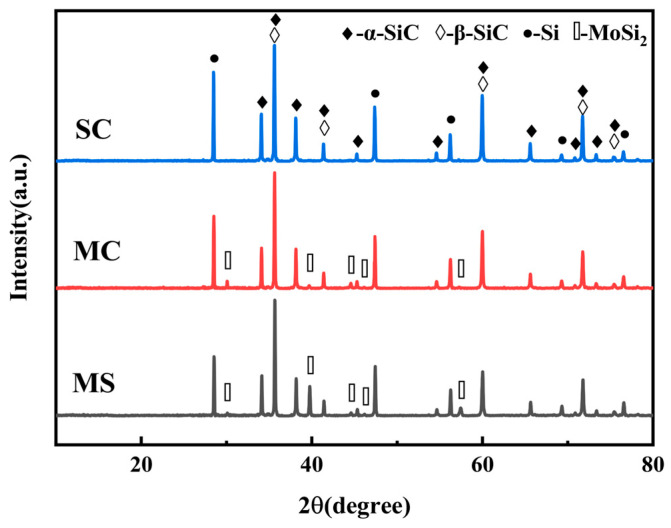
XRD patterns of SiC ceramic and SiC-MoSi_2_ composite samples after reactive sintering at 1600 °C for 2 h (SC, MC, and MS).

Figure 4 shows the X-ray diffraction patterns of the SiC ceramic and SiC-Mo_2_C composite samples after reactive sintering at 1600 °C for 2 h. In Sample SC, diffraction peaks corresponding to α-SiC (6H polytype), β-SiC (3C), and residual Si are identified, which is consistent with the typical phase composition of reaction-bonded SiC ceramics. The α-SiC originates from the starting raw material, while β-SiC is formed by the reaction between carbon black and molten silicon infiltrated into the porous SiC/C preform:

β-SiC is the low-temperature stable phase of SiC, and the sintering temperature of 1600 °C falls within the range favorable for its formation. The residual Si detected in Sample SC arises from excess molten silicon that fills the pores of the preform and remains unreacted. Quantitative analysis indicates that the residual Si content in Sample SC is as high as 19.17 ± 1.01 vol.% (Table 2).

Si(l) + C(s) → β-SiC(s)(7)

For Samples MC and MS, diffraction peaks of both α-SiC and β-SiC are also present. However, in contrast to Sample SC, two notable differences are observed: (i) the intensity of the Si diffraction peaks is significantly reduced, and (ii) additional peaks corresponding to MoSi_2_ appear. Both MC and MS contain Mo_2_C in their porous preforms. During reactive sintering, molten silicon infiltrates the SiC–Mo_2_C/C preform and reacts with Mo_2_C to form MoSi_2_ according to the following reaction:

Mo_2_C(s) + 5Si(l) = MoSi_2_(s) + β-SiC(s) (8)

Notably, compared to Sample MC, Sample MS exhibits even weaker Si diffraction peaks and stronger MoSi_2_ diffraction peaks. Quantitative analysis (Table 2) shows that the residual Si content decreases from 12.43 ± 0.86 vol.% in MC to 8.77 ± 0.45 vol.% in MS. This difference is attributed to the superior distribution uniformity of Mo_2_C within the green compact of Sample MS. The MS preform contains Mo_2_C/C composite powder synthesized by the molten salt method, which features finer Mo_2_C particles and a more homogeneous distribution compared to the physically mixed commercial Mo_2_C powder used in Sample MC. The finer and more uniform Mo_2_C particles provide a substantially larger contact area with the infiltrating molten silicon, kinetically promoting reaction (6). This enhanced reaction efficiency leads to the formation of more MoSi_2_ and a corresponding reduction in residual Si content.

Table 2 summarizes the densification results of the three samples. All samples achieve high densification, with open porosities below 0.1%. The bulk densities are 2.96 ± 0.05, 3.03 ± 0.03, and 3.07 ± 0.03 g/cm^3^ for Samples SC, MC, and MS, respectively. It is known that the theoretical densities of Si, SiC, and MoSi_2_ are 2.33, 3.21, and 6.28 g/cm^3^, respectively. Therefore, the decrease in residual Si content and the increase in MoSi_2_ content lead to higher sample densities, accounting for the observed density trend: SC < MC < MS.

**Figure 5:**
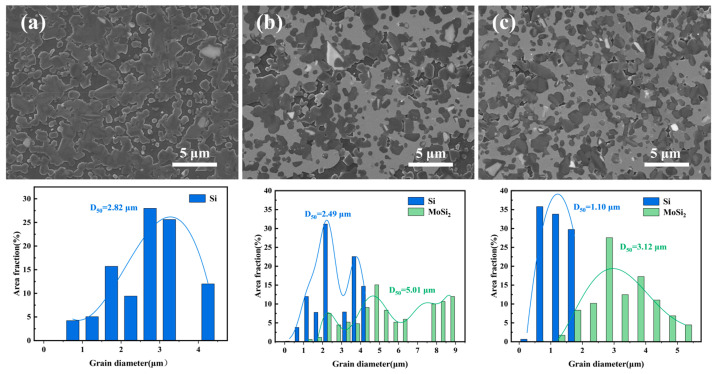
SEM images and corresponding particle size distribution maps of reactively sintered samples: (**a**) SC, (**b**) MC, (**c**) MS.

Figure 5 presents SEM images of the polished surfaces of the reactive sintered samples. All three samples exhibit highly dense microstructures, further confirming their high degree of densification. Different phases are distinguishable by their contrast levels, which arise from a mixed signal comprising secondary electrons and a minor contribution from backscattered electrons.

Sample SC (Figure 5a) consists of a continuous gray matrix composed of α-SiC and β-SiC, with discretely distributed dark regions corresponding to residual Si [51,52]. In contrast, Sample MC (Figure 5b) displays three distinct phases distinguishable by their contrast: bright gray MoSi_2_, light gray SiC, and minor dark regions of residual Si. Both the SiC and MoSi_2_ phases exhibit some degree of aggregation.

Sample MS (Figure 5c) contains the same three phases as Sample MC but exhibits significantly improved microstructural homogeneity. Quantitative statistical analysis of the size and distribution of residual Si and MoSi_2_ phases in the ceramic samples was performed using Image-Pro 6.0 software. The results show that the median particle size (D_50_) of residual Si in the MS sample is 1.10 μm, which is approximately 60% lower than that of the SC sample (2.82 μm). The D_50_ of MoSi_2_ in the MS sample is 3.12 μm, significantly smaller than that of the MC sample (5.01 μm), fully demonstrating that the phase sizes are effectively refined. Meanwhile, the particle size distributions of MoSi_2_ and residual Si in the MS sample are more concentrated, whereas the MC sample exhibits a broad and multi-modal distribution, indicating a higher homogeneity of phase distribution in the MS sample. Furthermore, the MC sample contains a large number of MoSi_2_ particles larger than 5 μm with a relatively high median particle size, providing direct quantitative evidence of obvious particle agglomeration. In contrast, the MS sample has no large agglomerated particles and exhibits better dispersion.

These microstructural observations further substantiate two key findings. First, the introduction of Mo_2_C into the preform effectively reduces the residual silicon content and promotes the formation of MoSi_2_ during reactive sintering. Second, the use of Mo_2_C/C composite powder synthesized by the molten salt method—characterized by finer Mo_2_C particles and more uniform distribution within the carbon black matrix—yields a more homogeneous microstructure and lower residual silicon content in the final reactive sintered product compared to the physically mixed commercial Mo_2_C powder.

### 3.3. Hydrothermal Corrosion Behavior

**Figure 6:**
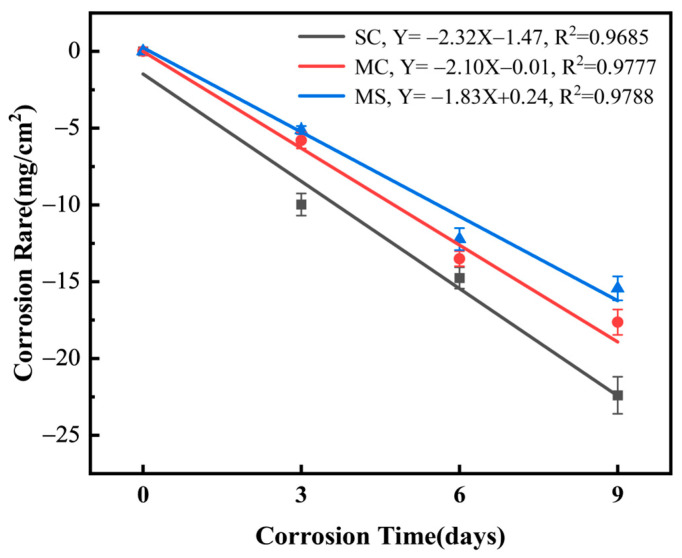
Fitted curves of corrosion weight change per unit area (∆*w*) for reactively sintered samples (SC, MC, MS) as a function of hydrothermal corrosion time at 345 °C/15 MPa.

Figure 6 illustrates the corrosion weight change per unit area (∆*w*) of samples (SC, MC, MS) as a function of hydrothermal corrosion time. As corrosion time extended from 3 to 9 days, ∆*w* remained negative, with weight loss increasing approximately linearly. The ∆*w* values at 3, 6, and 9 days were: SC (−9.9734 ± 0.7196, −14.7575 ± 0.6968, −22.3970 ± 1.2059 mg/cm^2^), MC (−5.7934 ± 0.5255, −13.5051 ± 0.4871, −17.6370 ± 0.8266 mg/cm^2^), and MS (−5.1261 ± 0.2634, −12.2270 ± 0.7194, −15.4347 ± 0.7807 mg/cm^2^). Consistently, weight loss followed SC > MC > MS during corrosion, confirming hydrothermal corrosion resistance in the order SC < MC < MS.

**Figure 7:**
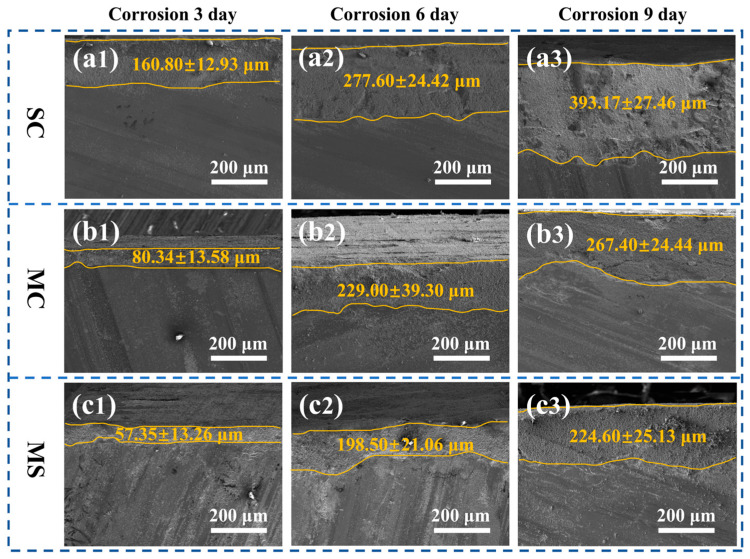
Cross-sectional SEM images of reactive sintered SiC and SiC–MoSi_2_ samples after hydrothermal corrosion: Sample SC after (**a1**) 3 days, (**a2**) 6 days, (**a3**) 9 days; Sample MC after (**b1**) 3 days, (**b2**) 6 days, (**b3**) 9 days; Sample MS after (**c1**) 3 days, (**c2**) 6 days, (**c3**) 9 days.

Cross-sectional morphologies of the samples after different corrosion durations were analyzed, and the corresponding SEM micrographs are shown in Figure 7. The microstructure can be divided into two distinct zones from top to bottom: a subsurface corrosion region characterized by a loose, porous structure, and an internal region exhibiting a relatively dense microstructure that remains largely unattacked.

As shown in Figure 7, the average corrosion depth (corresponding to the width of the porous subsurface region) of Samples SC, MC, and MS after 3, 6, and 9 days of hydrothermal corrosion is as follows: SC (160.80 ± 12.93, 277.60 ± 24.42, 393.17 ± 27.46 μm), MC (80.34 ± 13.58, 229.00 ± 39.30, 267.40 ± 24.44 μm), and MS (57.35 ± 13.26, 198.50 ± 21.06, 224.60 ± 25.13 μm). These results further confirm the hydrothermal corrosion resistance order: SC < MC < MS.

**Figure 8:**
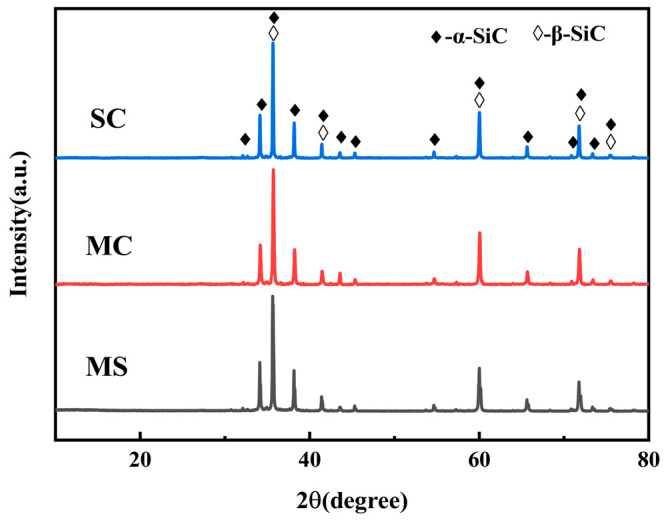
XRD patterns of the surfaces of reactive sintered SiC and SiC–MoSi_2_ samples after hydrothermal corrosion (345 °C, 15 MPa) for 9 days.

The XRD patterns of the corroded surfaces of reactive sintered SiC and SiC–MoSi_2_ samples after hydrothermal corrosion (345 °C, 15 MPa) for 9 days are shown in Figure 8. Only diffraction peaks corresponding to β-SiC and α-SiC are detected in all three samples. Compared to the pre-corrosion samples, the diffraction peaks of residual secondary phases, namely Si and MoSi_2_, have completely disappeared. It is reasonable to infer that these secondary phases were transformed into amorphous glassy phases (such as SiO_2_ and MoO_3_) during hydrothermal corrosion, which are not detectable by XRD [53]. Moreover, it is likely that a portion of these glassy phases dissolved into the aqueous medium, contributing to the observed weight loss.

### 3.4. Corrosion Mechanism and the Role of MoSi_2_

**Figure 9:**
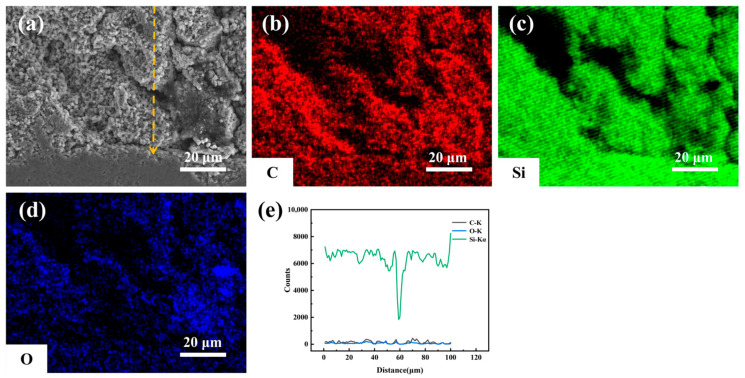
Microstructure and EDS elemental distribution of the corroded cross-section of SC samples after 9 days of hydrothermal corrosion. (**a**) Cross-sectional SEM image; (**b**) C elemental mapping; (**c**) Si elemental mapping; (**d**) O elemental mapping; (**e**) EDS line-scan profiles of C, O, and Si.

Figure 9 shows SEM-EDS analysis of the cross-sectional corrosion regions beneath the corroded surface of samples after 9 days of hydrothermal corrosion. Sample SC’s corrosion region exhibits obvious cracks and voids (some up to ~20 μm in diameter), consistent with its high weight loss (22.3970 ± 1.2059 mg/cm^2^). EDS elemental mapping reveals the corrosion region is mainly composed of Si, C, and O with highly coincident distributions, indicating it is predominantly SiC and SiO_2_. No distinct residual Si-enriched areas are detected, further confirming preferential dissolution and leaching of residual Si during corrosion. EDS line scans show a dominant Si signal across the entire cross-section (intrinsic to the SiC matrix), with line scan intensity representing relative counts rather than absolute atomic percentages.

**Figure 10:**
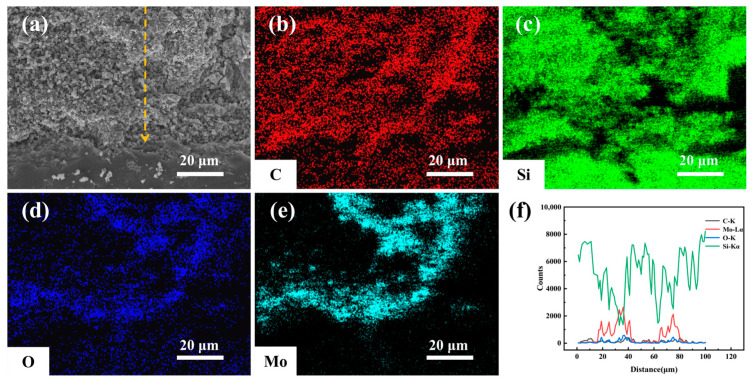
Microstructure and EDS elemental distribution of the corroded cross-section of MC samples after 9 days of hydrothermal corrosion: (**a**) Cross-sectional SEM image; (**b**) C elemental mapping; (**c**) Si elemental mapping; (**d**) O elemental mapping; (**e**) Mo elemental mapping; (**f**) EDS line-scan profiles of C, Mo, O, and Si.

Figure 10 presents SEM-EDS characterization of the cross-sectional corrosion region directly beneath Sample MC’s corroded surface after 9 days of hydrothermal corrosion. While the corrosion region remains porous overall, it differs significantly from Sample SC, featuring fewer cracks, smaller pores, and a denser structure. EDS elemental mapping shows the corrosion region mainly contains Si, C, O, and Mo, indicating the presence of uncorroded SiC matrix and Mo- and Si-containing oxide phases. Notably, Mo is more concentrated in the corrosion region, suggesting the added MoSi_2_ exerts a positive effect on enhancing hydrothermal corrosion resistance. In the 20–40 μm and 60–80 μm corrosion layers, Mo and O signals exhibit co-enrichment peaks, while Si remains dominant across the cross-section, primarily due to the intrinsic signal of the SiC matrix.

**Figure 11:**
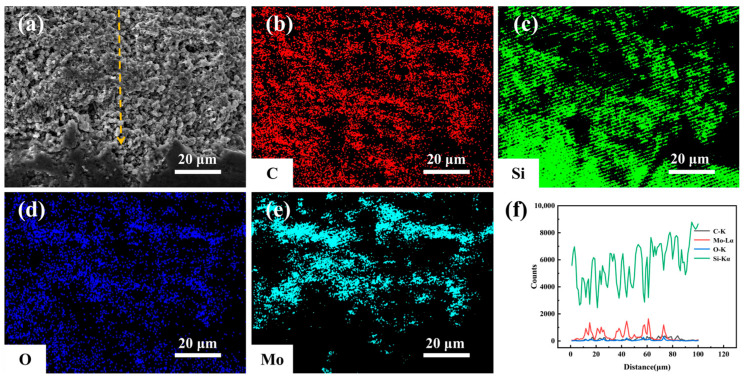
Microstructure and EDS elemental distribution of the corroded cross-section of MS samples after 9 days of hydrothermal corrosion: (**a**) Cross-sectional SEM image; (**b**) C elemental mapping; (**c**) Si elemental mapping; (**d**) O elemental mapping; (**e**) Mo elemental mapping; (**f**) EDS line-scan profiles of C, Mo, O, and Si.

Figure 11 shows SEM-EDS characterization of the cross-sectional corrosion region directly beneath Sample MS’s corroded surface after 9 days of hydrothermal corrosion. Among the three samples, Sample MS’s corrosion region has the lowest porosity and a more homogeneous microstructure. EDS mapping reveals the presence of Si, C, O, and Mo, with notably uniform Mo distribution—likely the key to its denser corrosion layer. The uniform MoSi_2_ distribution in Sample MS further enhances its positive effect on hydrothermal corrosion resistance. EDS line scans show distinct co-enrichment peaks of Mo and O (synchronized distribution) in the 10–80 μm corrosion region, confirming the formation of a MoO_3_-SiO_2_ composite oxide layer. The dominant Si signal across the cross-section is attributed to the intrinsic SiC matrix.

**Figure 12:**
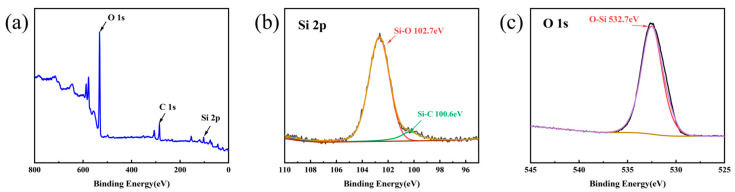
XPS analysis of the surface of Sample SC after hydrothermal corrosion for 9 days: (**a**) survey scan spectrum, (**b**) high-resolution Si 2p spectrum, (**c**) high-resolution O 1s spectrum.

X-ray photoelectron spectroscopy (XPS) was used to characterize the surface elemental distribution and chemical bonding states of Sample SC after 9 days of hydrothermal corrosion (Figure 12). The survey spectrum shows the surface is mainly composed of O, C, and Si (Figure 12a). Fitting of the high-resolution Si 2p spectrum identifies Si–O (102.7 eV) and Si–C (100.6 eV) bonds (Figure 12b) [54], while O 1s spectrum fitting confirms O–Si bonds (532.7 eV) (Figure 12c) [55,56], confirming SiO_2_ and SiC on the corroded surface. The absence of Si–Si bonds in the Si 2p spectrum indicates complete corrosion of the surface residual silicon.

**Figure 13:**
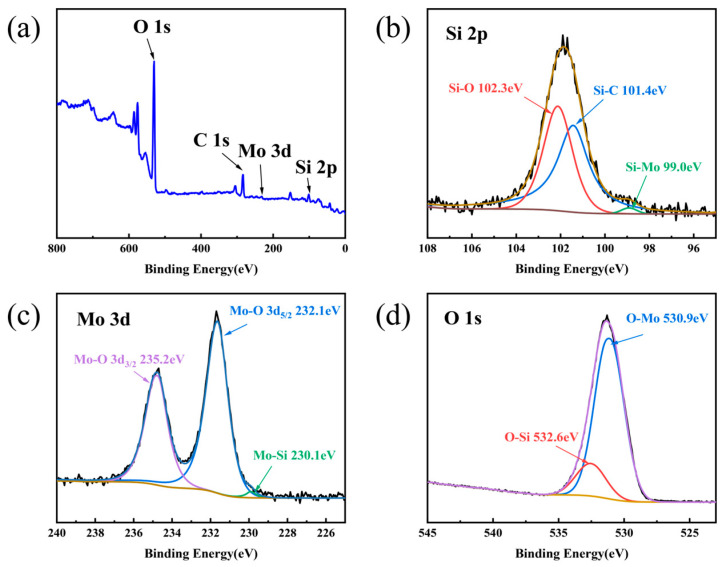
XPS analysis of the surface of Sample MC after hydrothermal corrosion for 9 days: (**a**) survey scan spectrum, (**b**) high-resolution Si 2p spectrum, (**c**) high-resolution Mo 3d spectrum, (**d**) high-resolution O 1s spectrum.

To investigate the corrosion behavior of the MC sample in a 345 °C, 15 MPa hydrothermal environment, XPS was used to analyze the surface elemental composition and chemical bonding states after 9 days of corrosion. Figure 13 shows the XPS survey spectrum and the high-resolution spectra of Si 2p, Mo 3d, and O 1s for the corroded surface. After 9 days of hydrothermal corrosion, the surface of MC-6 is mainly composed of O, C, Mo, and Si (Figure 13a). In Figure 13b, peak fitting of the high-resolution Si 2p spectrum reveals three components: Si–O bonds (102.3 eV), Si–C bonds (101.4 eV), and Si–Mo bonds (99.0 eV). In Figure 13c, peak fitting of the high-resolution Mo 3d spectrum identifies three characteristic peaks corresponding to Mo–O (3d_3/2_) bonds (235.2 eV), Mo–O (3d_5/2_) bonds (232.1 eV), and Mo–Si bonds (230.1 eV). In Figure 13d, the high-resolution O 1s spectrum is fitted into O–Si bonds and O–Mo bonds with binding energies of 532.6 eV and 530.5 eV, respectively [57,58,59,60,61]. Based on the above results, the presence of SiC, MoSi_2_, SiO_2_, and MoO_3_ can be confirmed on the corroded surface of the MC sample. Meanwhile, no Si–Si bonds are detected in the high-resolution Si 2p spectrum, indicating that the residual silicon on the sample surface has been completely corroded.

**Figure 14:**
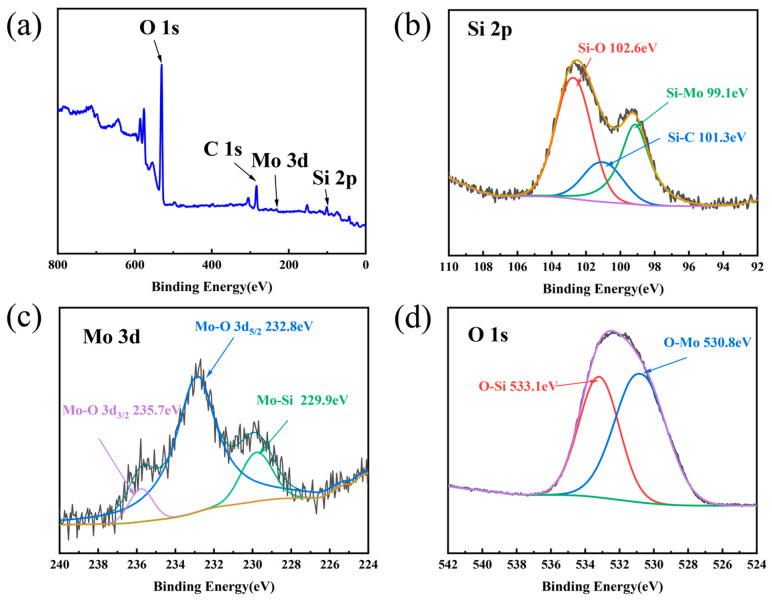
XPS analysis of the surface of Sample MS after hydrothermal corrosion for 9 days: (**a**) survey scan spectrum, (**b**) high-resolution Si 2p spectrum, (**c**) high-resolution Mo 3d spectrum, (**d**) high-resolution O 1s spectrum.

XPS was employed to reveal the elemental composition and chemical states on the surface of Sample MS after 9 days of hydrothermal corrosion. The survey scan spectrum (Figure 14a) displays characteristic peaks corresponding to Si 2p, Mo 3d, C 1s, and O 1s. The high-resolution Si 2p spectrum (Figure 14b) was deconvoluted into three peaks at binding energies of 99.1 eV, 101.3 eV, and 102.6 eV, assigned to Si–Mo, Si–C, and Si–O bonds, respectively. The high-resolution Mo 3d spectrum (Figure 14c) also comprises three peaks at 229.9 eV, 232.8 eV, and 235.7 eV, corresponding to Mo–Si, Mo–O (3d_3/2_), and Mo–O (3d_5/2_) bonds, respectively. The high-resolution O 1s spectrum (Figure 14d) is composed of two peaks at 530.8 eV and 533.1 eV, attributed to O–Mo and O–Si bonds, respectively. These results suggest that the corroded surface of Sample MS contains SiC, MoSi_2_, SiO_2_, and MoO_3_ [62,63,64,65,66]. Furthermore, the absence of a characteristic peak corresponding to Si–Si bonds indicates that no elemental silicon remains on the corroded surface.

Based on the above results, the three samples (SC, MC, and MS) exhibit distinctly different hydrothermal corrosion resistances, which are closely related to their composition and microstructure. Analysis of the residual phases in the corroded regions leads to the reasonable inference that the intrinsic hydrothermal corrosion resistance of the constituent phases follows the order Si < MoSi_2_ < SiC. During hydrothermal corrosion, the Si, MoSi_2_, and SiC constituents in the reactive sintered samples are likely oxidized via the following reactions [26,67,68]:

Si(s) + 2H_2_O(l) = SiO_2_(s) + 2H_2_(g) (9)

MoSi_2_(s) + 7H_2_O(l) = MoO_3_(s) + 2SiO_2_(s) + 7H_2_(g) (10)

SiC(s) + 3H_2_O(l) = SiO_2_(s) + CO(g) + 3H_2_(g) (11)

Subsequently, the SiO_2_ product may undergo further hydrothermal corrosion via the following reactions [69]:

SiO_2_(s) + H_2_O(l) = H_2_SiO_3_(aq) (12)

SiO_2_(s) + 2H_2_O(l) = Si(OH)_4_(aq) (13)

The products H_2_SiO_3_ and Si(OH)_4_ dissolve in water, forming weakly acidic solutions that can undergo ionization, rendering the corroded sample surface porous and loose.

As evident from the above chemical equations, residual silicon is ultimately completely consumed by hydrothermal corrosion, with its products (H_2_SiO_3_ and Si(OH)_4_) dissolving in the aqueous medium, thereby creating pores within the corrosion region. This allows the ingress of high-temperature, high-pressure water further into the sample, continuing the corrosion process. In contrast, MoSi_2_ is oxidized to MoO_3_, which has extremely low solubility in high-temperature and high-pressure aqueous environments and can stably remain in the corrosion layer [69,70]. The low-solubility MoO_3_ exerts a pinning effect on soluble SiO_2_, immobilizing it in the oxide film and suppressing its excessive dissolution and loss. This maintains a relatively dense microstructure, protecting the underlying uncorroded matrix from further hydrothermal corrosion. Thus, Mo significantly improves the compactness and structural stability of the MoO_3_-SiO_2_ composite oxide layer, inhibits its further dissolution into highly soluble H_2_SiO_3_ and Si(OH)_4_, and ultimately enhances the material’s hydrothermal corrosion resistance.

**Figure 15:**
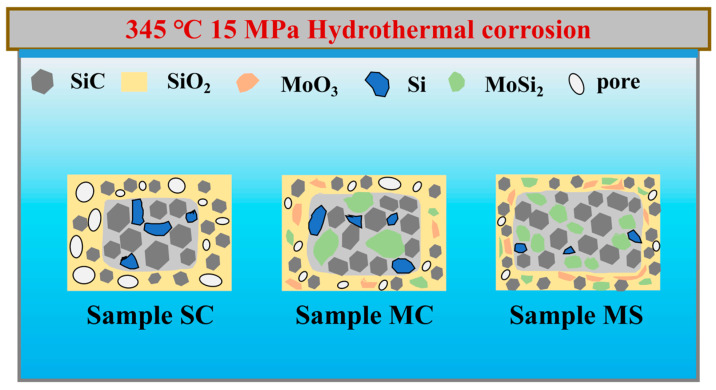
Schematic structure diagram of the hydrothermal corrosion mechanism of SC, MC, and MS samples.

A comparison of the hydrothermal corrosion mechanisms of the three samples is schematically illustrated in Figure 15. From a microstructural perspective, the SC sample is characterized by a high (19.17 ± 1.01 vol.%) and continuously distributed residual silicon phase. During the initial stage of hydrothermal corrosion, its surface is corroded, forming through-penetrating macropores. As corrosion proceeds, a dynamic cycle of “continuous reaction–pore expansion” is established, ultimately resulting in the highest weight loss and deepest corrosion depth.

For Sample MC, the residual silicon content is reduced to 12.43 ± 0.86 vol.%, and MoSi_2_ is present as a secondary phase. However, its microstructure is insufficiently uniform: the presence of large residual Si blocks and unevenly distributed MoSi_2_ still limits the improvement in corrosion resistance. The larger residual Si regions are still susceptible to hydrothermal corrosion, forming large-sized pore channels that allow further water ingress. Moreover, the larger size and uneven distribution of MoSi_2_ hinder the formation of a continuous and uniformly distributed MoO_3_-SiO_2_ composite oxide film. Consequently, although its hydrothermal corrosion resistance surpasses that of Sample SC, the optimal improvement is not yet achieved.

Sample MS has the lowest residual silicon content (8.77 ± 0.45 vol.%) and fine, uniformly distributed MoSi_2_. The pores generated after hydrothermal corrosion of Si are very small; meanwhile, the fine, uniformly distributed MoSi_2_ forms a continuous, homogeneous MoO_3_-SiO_2_ composite oxide protective film via hydrothermal oxidation. Owing to MoO_3_’s much lower hydrothermal solubility, it acts as a pinning agent to retain soluble SiO_2_ in situ, preventing excessive dissolution and maintaining the oxide film’s compactness and stability. These two factors (minimized pore formation and effective protective barrier) synergistically block high-temperature, high-pressure water ingress, protecting the internal material from further corrosion—thus, Sample MS exhibits the lowest weight loss and shallowest corrosion depth.

Our findings demonstrate that modifying traditional RBSC materials by introducing fine and uniformly distributed MoSi_2_ while simultaneously reducing the residual silicon content effectively enhances hydrothermal corrosion resistance.

## 4. Conclusions

In this study, reaction-bonded SiC–MoSi_2_ composites were fabricated with the aim of enhancing the hydrothermal corrosion resistance of conventional reaction-bonded SiC ceramics through the introduction of MoSi_2_ and the reduction in residual silicon content. Three material systems, all prepared by reactive melt infiltration, were comparatively investigated, with the primary distinction lying in the composition and preparation route of the preforms: (i) conventional RBSC with a SiC/C preform (SC); (ii) a SiC–MoSi_2_ composite incorporating commercial Mo_2_C powder via physical mixing to obtain a SiC/Mo_2_C/C preform (MC); and (iii) a SiC–MoSi_2_ composite derived from a Mo_2_C/C precursor synthesized by a molten salt method, subsequently mixed with SiC powder to form the preform (MS). The microstructural evolution, phase composition, and hydrothermal corrosion behavior of each system were systematically evaluated.

(1) The molten salt synthesis at 1150 °C yielded a Mo_2_C/C composite powder characterized by fine and uniformly distributed Mo_2_C particles intimately coated on carbon black. This contrasted sharply with the agglomerated and inhomogeneous distribution observed in the physical mixture of commercial Mo_2_C and carbon black. During reactive sintering at 1600 °C, the Mo_2_C component in both MC and MS preforms reacted with infiltrated molten silicon to form MoSi_2_, effectively reducing the residual silicon content. The MS sample delivered the lowest residual silicon content (8.77 ± 0.45 vol.%), followed by MC (12.43 ± 0.86 vol.%) and SC (19.17 ± 1.01 vol.%). Correspondingly, the bulk density increased from 2.96 ± 0.05 g/cm^3^ for SC to 3.03 ± 0.03 g/cm^3^ for MC and 3.07 ± 0.03 g/cm^3^ for MS, with all samples achieving near-full densification (open porosity < 0.1%).
(2) From a microstructural perspective, fine MoSi_2_ grains were homogeneously distributed throughout the SiC matrix in Sample MS. In contrast, Sample MC suffered from partial aggregation of MoSi_2_ alongside residual Si, while continuous unreacted silicon regions prevailed in Sample SC. Such distinct microstructural features governed the hydrothermal corrosion performance. Hydrothermal evaluations at 345 °C and 15 MPa over 9 days confirmed a corrosion resistance trend of MS > MC > SC. After long-term exposure, the areal weight losses of SC, MC and MS were 22.3970 ± 1.2059 mg/cm^2^, 17.6370 ± 0.8266 mg/cm^2^ and 15.4347 ± 0.7807 mg/cm^2^, with corresponding corrosion depths of 393.17 ± 27.46 μm, 267.40 ± 24.44 μm and 224.60 ± 25.13 μm, respectively.
(3) The enhanced performance of Sample MS is attributed to two synergistic factors. First, the substantial reduction in residual silicon content minimized the formation of large corrosion-induced pores that would otherwise serve as pathways for further water ingress. Second, the uniform distribution of fine MoSi_2_ grains facilitates the formation of a continuous and stable composite oxide layer of MoO_3_-SiO_2_ during hydrothermal oxidation. In this protective layer, low-solubility MoO_3_ exerts a pinning effect on SiO_2_, immobilizing it in situ and restraining its excessive dissolution. This layer exhibits superior resistance to dissolution compared to the SiO_2_ layer formed on unmodified RBSC, thereby effectively mitigating further corrosion of the underlying material. In contrast, the inhomogeneous distribution of MoSi_2_ and larger residual Si domains in Sample MC limited its protective efficiency, while the complete absence of MoSi_2_ in Sample SC rendered it most susceptible to hydrothermal attack.
(4) These results demonstrate that the combination of residual silicon minimization and homogeneous incorporation of MoSi_2_—achieved through the use of a molten salt-derived Mo_2_C/C precursor in the preform—offers an effective and promising strategy for substantially improving the hydrothermal corrosion resistance of reaction-bonded SiC-based materials. It provides practical guidance for the microstructure regulation and compositional optimization of conventional RBSC fabrication processes. These findings hold significant promise for advancing the application of melt-infiltrated SiC*_f_*/SiC composites as accident-tolerant fuel cladding materials in nuclear water reactors, where superior corrosion resistance in high-temperature and high-pressure aqueous environments is critically required.

## Figures and Tables

**Table 1:** EDS point analysis results (at.%) for the marked regions in the SEM images shown in Figure 3.

Position	Mo	C
Point 1	32.3	67.7
Point 2	8.2	91.8
Point 3	10.7	89.3
Point 4	2.3	97.7

**Table 2:** Residual Si contents and densification results of SC, MC, and MS samples after reactive sintering at 1600 °C for 2 h.

Sample	Residual Si Content (vol.%)	Bulk Density (g/cm^3^)	Open Porosity (%)
SC	19.17 ± 1.01	2.96 ± 0.05	0.05 ± 0.02
MC	12.43 ± 0.86	3.03 ± 0.03	0.03 ± 0.01
MS	8.77 ± 0.45	3.07 ± 0.03	0.02 ± 0.02

## Data Availability

The data presented in this study are available on request from the corresponding author due to privacy and legal reasons.

## References

[1] Lu Y.-H., Zhang J.-Z., Li G.-Q., Wang Z.-H., Wu J., Wei C. (2025). A Review of Joining Technologies for SiC Matrix Composites. Materials.

[2] Liu Z.-F., Chen X.-J., Chen B.-X., Hu Y.-M., Zheng K.-Y., Liu G.-Z., Chen A.-N., Chen P., Li Z.-Q., Yang L. (2026). The Mechanical Enhancement Mechanism in Additively Manufactured Continuous SiC*_f_*/SiC Ceramic Matrix Composites. J. Eur. Ceram. Soc..

[3] Yang X., Huang Q.-Z., Su Z.-A., Chang X., Chai L.-Y., Liu C.-X., Xue L., Huang D. (2013). Resistance to Oxidation and Ablation of SiC Coating on Graphite Prepared by Chemical Vapor Reaction. Corros. Sci..

[4] Wang P.-P., Li H.-J., Jia Y.-J., Zhang Y.-L., Yuan R.-M. (2017). Ablation Resistance of HfB_2_-SiC Coating Prepared by in-Situ Reaction Method for SiC Coated C/C Composites. Ceram. Int..

[5] Wang B., Li G., Li J.-Y., Wang L., Zhuang X.-P., Shi W., Tan Y. (2023). Interfacial Modification and Oxidation Resistance Behavior of a CVD-SiC Coating for C/SiC Composites. Ceram. Int..

[6] Wang Z.-Y., De Y.-Y., Wei C., Zhang S.-B., Li X.-Q. (2025). Corrosion Behavior of NITE-SiC with Different Sintering Additives at Simulated PWRs Conditions. Ceram. Int..

[7] Huczko A., Dabrowska A., Savchyn V., Popov A.I., Karbovnyk I. (2009). Silicon carbide nanowires: Synthesis and cathodoluminescence. Phys. Status Solidi B.

[8] Tynyshbayeva K.M., Kozlovskiy A.L. (2025). The effect of temperature factor during heavy ion irradiation on structural disordering of SiC ceramics. Opt. Mater. X.

[9] Lebedev A.S., Suzdaltsev A.V., Anfilogov V.N., Farlenkov A.S., Porotnikova N.M., Vovkotrub E.G., Akashev L.A. (2020). Carbothermal Synthesis, Properties, and Structure of Ultrafine SiC Fibers. Inorg. Mater..

[10] Katoh Y., Nozawa T., Snead L.L., Ozawa K., Tanigawa H. (2011). Stability of SiC and Its Composites at High Neutron Fluence. J. Nucl. Mater..

[11] Kishimoto H., Katoh Y., Kohyama A. (2002). Microstructural Stability of SiC and SiC/SiC Composites under High Temperature Irradiation Environment. J. Nucl. Mater..

[12] Snead L.L., Nozawa T., Katoh Y., Byun T.-S., Kondo S., Petti D.A. (2007). Handbook of SiC Properties for Fuel Performance Modeling. J. Nucl. Mater..

[13] Lee Y., No H.C., Lee J.I. (2017). Design Optimization of Multi-Layer Silicon Carbide Cladding for Light Water Reactors. Nucl. Eng. Des..

[14] Agarwal A. (2025). Structural analysis of helical coil suspension using direct optimization technique for enhanced performance. AIP Conf. Proc..

[15] Cao X.-Q., Wang J.-J., Liang Y.-M., Zhang G.-A., Shang L.-L., Lu Z.-B., Xue Q.-J. (2020). Corrosion and tribological investigations of the B_4_C coatings rubbing against SiC ball for high relative humidity engineering application. Mater. Today Commun..

[16] Hassan R., Balani K. (2020). Engineered Role of SiC Particle Size on Multi-Length-Scale Wear Damage of Spark Plasma Sintered Zirconium Diboride. Adv. Eng. Mater..

[17] Huang C.-C., Chen J., Zhu M., Li F.-F., Liu X.-J., Huang Z.-R. (2023). Two-Step Joining of Reaction Bonded Silicon Carbide (RBSC) Using Borosilicate Glass. J. Mater. Res. Technol..

[18] Margiotta J.C., Zhang D., Nagle D.C. (2010). Microstructural Evolution during Silicon Carbide (SiC) Formation by Liquid Silicon Infiltration Using Optical Microscopy. Int. J. Refract. Met. Hard Mater..

[19] Hsu C., Zhang Y., Xie Y., Deng F., Karandikar P., Xiao J.Q., Ni C. (2019). In-Situ Measurement of SiC/Si Interfacial Tensile Strength of Reaction Bonded SiC/Si Composite. Compos. Part B Eng..

[20] Huang Q.-W., Zhu L.-H. (2005). High-Temperature Strength and Toughness Behaviors for Reaction-Bonded SiC Ceramics below 1400 °C. Mater. Lett..

[21] Song N., Zhang H.-B., Liu H., Fang J.-Z. (2017). Effects of SiC Whiskers on the Mechanical Properties and Microstructure of SiC Ceramics by Reactive Sintering. Ceram. Int..

[22] Lee J., Kim D., Shin D., Lee H.G., Park J.Y., Kim W.J. (2021). A new process for minimizing residual silicon and carbon of reaction-bonded silicon carbide via chemical vapor deposition. J. Eur. Ceram. Soc..

[23] Grinchuk P.S., Abuhimd H.M., Kiyashko M.V., Solovei D.V., Akulich A.V., Stepkin M.O., Evseeva L.E. (2024). Advanced reaction-bonded SiC ceramics for space mirror blanks. J. Manuf. Process..

[24] Chakrabarti O.P., Ghosh S., Mukerji J. (1994). Influence of grain size, free silicon content and temperature on the strength and toughness of reaction-bonded silicon carbide. Ceram. Int..

[25] Ren Q.-X., Yin Z.-Q., Guo X.-Y., Chun S.-X., Nie H.F., Yan S., Ru H.-Q., Huang Z.-R., Huang Q., Li Y.-S. (2026). Enhanced thermal conductivity of reaction-bonded silicon carbide via constructing a mesoporous carbon network in preform. J. Mater. Sci. Technol..

[26] Kim W.-J., Hwang H.-S., Park J.-Y. (2002). Corrosion Behavior of Reaction-Bonded Silicon Carbide Ceramics in High-Temperature Water. J. Mater. Sci. Lett..

[27] Xi J.-Q., Liu C., Morgan D., Szlufarska I. (2021). Deciphering Water-Solid Reactions during Hydrothermal Corrosion of SiC. Acta Mater..

[28] He F., Liu Y.-S., Li J.-X., Liu Q.-M., Cao Y.-J., Wang J., Dong N. (2024). The Impact of Water and Oxygen Contents on the Corrosion Performance of Yttrium Silicate Modified SiC*_f_*/SiC Composites under High Temperature Conditions. J. Eur. Ceram. Soc..

[29] Aroati S., Cafri M., Dilman H., Dariel M.P., Frage N. (2011). Preparation of reaction bonded silicon carbide (RBSC) using boron carbide as an alternative source of carbon. J. Eur. Ceram. Soc..

[30] Xue R., Liu P., Zhang Z.-J., Zhang N.-L., Zhang Y.-H., Wang J.-P. (2021). Improvement of toughness of reaction bonded silicon carbide composites reinforced by surface-modified SiC whiskers. Ceram. Int..

[31] Li W., Cui C.-C., Li S., Zhang G., Jin B.-J., Bao J.-X., Guo C.-H., Zhang Y.-B., Liu B.-S., Wang G. (2024). Vat photopolymerization of large-aperture high performance SiC mirror through multiphase carbon infiltration modification. Addit. Manuf..

[32] Zhang H.-Z., Zhu Z.-B., Huang H.-F., He T., Yan H.-W., Zhang Y.-G., Lu Y.-P., Wang T.-M., Li T. (2023). Microstructures, Mechanical Properties, and Irradiation Tolerance of the Ti–Zr–Nb–V–Mo Refractory High-Entropy Alloys. Intermetallics.

[33] Zhu Z.-B., Huang H.-F., Muránsky O., Liu J.-Z., Zhu Z.-Y., Huang Y. (2021). On the Irradiation Tolerance of Nano-Grained Ni–Mo–Cr Alloy: 1 MeV He+ Irradiation Experiment. J. Nucl. Mater..

[34] Daydas S., Tiftikci A. (2024). Neutronic Examination of the U–Mo Accident Tolerant Fuel for VVER-1200 Reactors. Nucl. Eng. Technol..

[35] Zhang Z.-J., Han E.-H., Xiang C. (2021). Irradiation Behaviors of Two Novel Single-Phase Bcc-Structure High-Entropy Alloys for Accident-Tolerant Fuel Cladding. J. Mater. Sci. Technol..

[36] Ge X.-X., Jiang Y.-X., Yu X., Zhang G.-P., Shi Y.-J., Cai B., Peng Q., Huang H. (2025). Preparation and Characterization of Graphene-Nanosheet-Reinforced Ni-17Mo Alloy Composites for Advanced Nuclear Reactor Applications. Materials.

[37] Vasudévan A.K., Petrovic J.J. (1992). A Comparative Overview of Molybdenum Disilicide Composites. Mater. Sci. Eng. A.

[38] Petrovic J.J. (1995). Mechanical Behavior of MoSi_2_ and MoSi_2_ Composites. Mater. Sci. Eng. A.

[39] Chang Q., Zhang Q., Xia S.-R., Liu C.-D., Cheng L.-F., Yu J. (2026). Microstructure Evolution of the 5 MeV Xe^20+^-Implanted α-MoSi_2_ before and after Annealing. Ceram. Int..

[40] Zhu L., Zhu N.-N., Tang P.-J., Zhang B.-J., Lei S.-Y., Wang X.-H., Feng P.-Z. (2026). Dual Strategy to Enhance the Nitridation Resistance of MoSi_2_-Based Ceramics for High-Temperature Applications. J. Eur. Ceram. Soc..

[41] Lv Y.-L., Liu Y.-S., Zhang B.-H., Liu M.-Z., Fu S.-L., Cao Y.-J., Li J.-X., Liu Y.-S., Pedzich Z. (2026). Novel MoSi_2_-Modified HfB_2_-SiC Composites Fabricated by Reactive Melt infiltration: Microstructure and Long-Term Oxidation Resistance at 1650 °C. Corros. Sci..

[42] Sun Y., Zheng L., Zhang S., Luo X., Liu F., Li Y. (2022). In Situ Synthesis of Al_2_O_3_–SiC Powders via Molten-Salt-Assisted Aluminum/Carbothermal Reduction Method. Ceram. Int..

[43] Yan M.G., Xiong Q.-M., Huang J.-T., Hou X.-F., Zhang L., Li X.-B., Feng Z.-J. (2021). Molten Salt Synthesis of Titanium Carbide Using Different Carbon Sources as Templates. Ceram. Int..

[44] He M.-X., Dong M.-L., Wang C.-H., You Y., Liu P.-W. (2025). Preparation of High Purity Ti_3_SiC_2_ MAX Phase from Ti, Si, and TiC Powders by Molten Salt Method. Ceram. Int..

[45] Li F.-L., Tan C., Liu J.-H., Wang J.-K., Jia Q.-L., Zhang H.-J., Zhang S.W. (2019). Low Temperature Synthesis of ZrB_2_-SiC Powders by Molten Salt Magnesiothermic Reduction and Their Oxidation Resistance. Ceram. Int..

[46] Yu L.-L., Hu Z.-L., Wang Z.-Z., Wang H. (2025). The effect of NaOH on the morphology of dislocation-related pits in SiC during etching in molten eutectic KOH-NaOH. Mater. Sci. Semicond. Process..

[47] Winterhalter F., Medri V., Ruffini A., Bellosi A. (2004). Corrosion of Si_3_N_4_-MoSi_2_ ceramic composite in acid-and basic-aqueous environments: Surface modification and properties degradation. Appl. Surf. Sci..

[48] Striegler M., Matthey B., Mühle U., Michaelis A., Herrmann M. (2018). Corrosion resistance of silicon-infiltrated silicon carbide (SiSiC). Ceram. Int..

[49] Hussain S., Zaidi S.A., Vikraman D., Kim H.-S., Jung J. (2019). Facile preparation of molybdenum carbide (Mo_2_C) nanoparticles and its effective utilization in electrochemical sensing of folic acid via imprinting. Biosens. Bioelectron..

[50] Etebarian S., Sarpoolaky H., Rezaie H.R., Velashjerdi M. (2023). Synthesis of Ti_3_SiC_2_ MAX Phase Powder through Molten Salt Method. Int. J. Appl. Ceram. Technol..

[51] Xu J.-L., Du S.-Y., Zhang Y.-M. (2025). The Molecular Dynamic Studies of Thermal Conductivity of SiC Ceramic Derived from β/α Phase Transformation. Sci. Rep..

[52] Shao Q.-Q., Gu H. (2023). Transition-Layer of Core–Rim Structures and β→α Transformation in SiC Ceramics. J. Mater..

[53] Ge F.-F., Zhou M.-T., Li B.-S., Yu P., Yan Z.-F., Cui M.-H., Shen T.-L. (2025). Effect of the 1.52 MeV Proton Irradiation on Hydrothermal Corrosion of Sintered SiC. Corros. Sci..

[54] Motailo E.S., Lisyanskii L.A., Vikhman S.V., Nesmelov D.D. (2021). Physical and Mechanical Properties of Composite Ceramics in the ZrB_2_-SiC-MoSi_2_ System. Glass Phys. Chem..

[55] Zhang W.-W., Li B.-S. (2018). Electrochemical Properties and XPS Analysis of Ni-B/SiC Nanocomposite Coatings. Int. J. Electrochem. Sci..

[56] Jin H., Meng S.-H., Zhang X.-H., Zeng Q.-X., Niu J.-H. (2016). Effects of oxygen partial pressure on the oxidation of ZrB_2_-SiC-graphite composites at 1800 °C. Ceram. Int..

[57] Ma Q.-Z., Hu R.-H., Liu X.-C., Lu X.-H., Chen J. (2026). Interfacial adhesion of CVD-SiC coatings on graphite substrates: Role of temperature in microstructure and mechanical properties. Ceram. Int..

[58] Chen D., Gu H.-Z., Huang A., Ni H.-W. (2019). Towards chrome-free lining for plasma gasifiers using the CA6-SiC castable based on high-temperature water vapor corrosion. Ceram. Int..

[59] Wang L., Wang S.-Y. (2025). Quantitative analysis of self-healing properties and microstructure of Ti-5Al-5Mo-5V-1Cr-1Fe alloy by quasi-in-situ XPS. J. Alloys Compd..

[60] Zhao H., Xie L.-F., Xin C., Li N., Zhao B., Li L.-Y. (2023). Effect of molybdenum content on corrosion resistance and corrosion behavior of Ti-Mo titanium alloy in hydrochloric acid. Mater. Today Commun..

[61] Zhang B.-J., Liu Z., Cheng J.-S., Yu L., Zhang Z.-X., Li J.-X., Li S.-H., Feng P.-Z. (2025). In situ synthesis of Fe-Mo alloys via synergistic smelting of copper slag and spent MoSi_2_ rods: Phase transformation and decomposition mechanism of MoSi_2_. J. Mater. Res. Technol..

[62] Liu J.-X., Wang N., Sun Y.-B., Yan X.-B., Wei S., Kang L., Du B.-X., Yang W.-W., Ma K. (2025). Ability of a MoSi_2_ Coating to Resist Erosion-Corrosion from the Impact of Atomic Oxygen. Ceram. Int..

[63] Lima C.D.A., De Carvalho T.C.V., Mendoza C.D., Maia Da Costa M.E.H., Pinheiro G.D.S., Luz-Lima C., Silva B.G., Sommer R.L., Araujo J.F.D.F. (2025). Magnetic Transition in MoO_3_: Influence of Mo^5+^/Mo^6+^ Ratios on Paramagnetic to Diamagnetic Behavior. Solid State Sci..

[64] Qin Y.-M., Li X.-Q., Liu C.-X., Zheng C., Mao Q.-P., Chen B., Jing K., Tan Y.-R., Cheng L.-F., Zhang L.-T. (2021). Effect of Deposition Temperature on the Corrosion Behavior of CVD SiC Coatings on SiC*_f_*/SiC Composites under Simulated PWR Conditions. Corros. Sci..

[65] Yao Z., Stiglich J., Sudarshan T.S. (1999). Molybdenum Silicide Based Materials and Their Properties. J. Mater. Eng. Perform..

[66] Alov N.V. (2015). XPS Study of MoO_3_ and WO_3_ Oxide Surface Modification by Low-energy Ar^+^ Ion Bombardment. Phys. Status Solidi C.

[67] Yang H., Li X.-Q., Liu C.-X., Zhao Y.-H., Chen B., Yang X., Cheng L.-F., Zhang L.-T. (2018). Hydrothermal Corrosion Behavior of SiC*_f_*/SiC Composites Candidate for PWR Accident Tolerant Fuel Cladding. Ceram. Int..

[68] Sooby Wood E., Parker S.S., Nelson A.T., Maloy S.A. (2016). MoSi_2_ Oxidation in 670–1498 K Water Vapor. J. Am. Ceram. Soc..

[69] Terrani K.A., Yang Y., Kim Y.-J., Rebak R., Meyer H.M., Gerczak T.J. (2015). Hydrothermal Corrosion of SiC in LWR Coolant Environments in the Absence of Irradiation. J. Nucl. Mater..

[70] Shang L.-B., Williams-Jones A.E., Wang X.-S., Timofeev A., Hu R.-Z., Bi X.-W. (2020). An experimental study of the solubility and speciation of MoO_3_(s) in hydrothermal fluids at temperatures up to 350 °C. Econ. Geol..

